# Small-molecule activation of NAMPT as a potential neuroprotective strategy

**DOI:** 10.1093/lifemedi/lnac012

**Published:** 2022-06-28

**Authors:** Xuanyu Gu, Hong Yao, Ilmin Kwon, Gelin Wang

**Affiliations:** School of Pharmaceutical Sciences, Beijing Advanced Innovation Center for Structural Biology, Ministry of Education Key Laboratory of Bioorganic Phosphorus Chemistry and Chemical Biology, Tsinghua University, Beijing 100084, China; School of Pharmaceutical Sciences, Beijing Advanced Innovation Center for Structural Biology, Ministry of Education Key Laboratory of Bioorganic Phosphorus Chemistry and Chemical Biology, Tsinghua University, Beijing 100084, China; Department of Anatomy and Cell Biology, Sungkyunkwan University School of Medicine, Suwon 16419, Korea; School of Pharmaceutical Sciences, Beijing Advanced Innovation Center for Structural Biology, Ministry of Education Key Laboratory of Bioorganic Phosphorus Chemistry and Chemical Biology, Tsinghua University, Beijing 100084, China

Neurodegenerative disorders comprise a wide array of conditions that result from progressive damage to nerve cells and nervous system connections, affecting an increasing number of patients, while so far, no effective treatments exist for most of these complex neurological diseases. Progress in the development of medicine to harness different individual diseases has been slow in the last half-century. In this context, strategies for targeting common features in a wide range of neurodegenerative diseases may be plausible.

Nicotinamide adenine dinucleotide (NAD) is a fundamental metabolite in many cellular processes by function as a cofactor in numerous redox reactions or as a signaling molecule through NAD-consuming enzymes. Decreased NAD level has not only been observed in physiological brain aging, but also closely associated with the progression of neurodegenerative diseases including Alzheimer’s disease (AD), Parkinson’s disease (PD), and amyotrophic lateral sclerosis (ALS), participating in almost all hallmarks of neuronal aging [1]. While the cellular processes and detailed mechanisms about how NAD is involved remain unknown, there is emerging evidence that NAD metabolite is important in neuroprotection and neurological health maintenance. The first such evidence came from a mutant resistant to axonal degeneration, known as Wallerian degeneration slow (WldS). WldS mice have been found to express an axon-located chimeric fusion protein of NAD-synthesizing enzyme nicotinamide mononucleotide adenylyltransferases 1 (NMNAT1), which replace the function of NMNAT2 that would otherwise be degraded after disruption of axonal protein transportation [2]. The second evidence came from a forward genetic screen in *Drosophila* [3], which identified sterile alpha and TIR motif-constraining 1 (SARM1) mutants having similar properties to that of the WldS mutation in mice. Subsequent extensive studies revealed that SARM1 is a *bona fide* NAD-consuming enzyme, and the physical destruction of axons leading to Wallerian degeneration is executed by activated SARM1 degrading NAD [4]. These studies imply the potential for neuroprotection by modulating NAD levels through the manipulation of enzymes involved in NAD metabolism.

The third evidence also came from an unbiased study, but a pharmacological screen approach has been used instead. Aiming toward the discovery of pro-neurogenic chemicals, phenotype-based screening in live mice identified an aminopropyl carbazole small molecule denoted as P7C3, which efficiently promotes neurogenesis in the hippocampus and enhances cognition. Then, through chemical optimization, P7C3-A20 and P7C3-S243 have been identified as more active analogs, showing efficacy in a variety of animal models of neurodegeneration [5]. To elucidate the mode of action of P7C3, researchers designed a P7C3 derivative P7C3-S326 as a CLICK chemistry probe, which can be covalently linked to its target after photo-crosslinking. By a competition binding assay of the probe against the original P7C3-A20, a protein component that could be competitively probed is obtained. The following proteomics studies revealed NAMPT as a selective binding protein for P7C3. This was in turn confirmed by in vivo and in vitro NAD level elevation after P7C3 treatment [6]. These studies, originating from a neuroprotective phenotype, demonstrated the feasibility of targeting the NAD biosynthetic enzyme NAMPT to promote NAD levels for neuroprotective functions.

**Figure 1.**
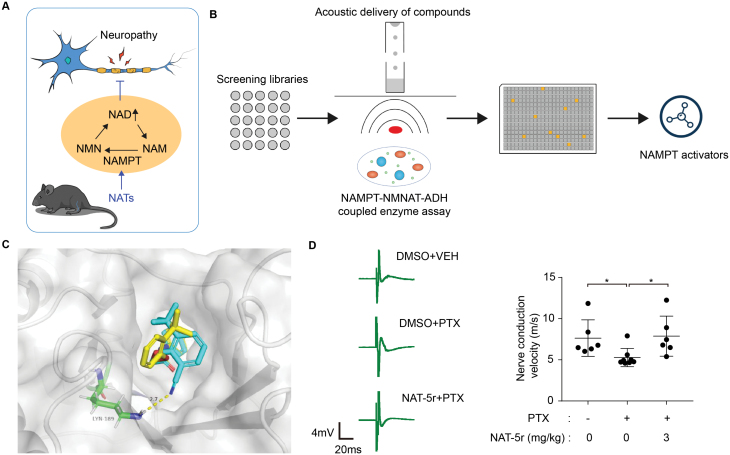
Small molecules NATs targeting NAD salvage enzyme NAMPT for neuroprotection. (A) Schematic mechanism of action of NATs in CIPN mouse models to relieve neuropathy. (B) Schematic for the procedure of the high-throughput screening for NAMPT activators. NMN produced by NAMPT was converted to NADH in a triply coupled enzyme assay and could be detected by fluorescence measurement (Ex_340_/Em_445_). (C) Crystal structure of purified, recombinant NAMPT in complex with NAT and docking model of NAMPT-NAT-5r complex. NAMPT is presented in gray. NAT and NAT-5r are shown in yellow and blue, respectively. Dotted lines indicate hydrogen bonds. (D) Sciatic nerve conduction velocity (NCV) of adult male C57BL/6J mice treated with vehicle or 3 mg/kg NAT-5r in combination with vehicle or PTX. Left panel, representative recording of nerve action potentials. Right panel, the mean NCV. Each symbol represents data from an individual mouse (*n* = 6–8). Bars represent means ± SEM.

Intuitively, two ideas to elevate NAD levels are to control the activity of NAD synthesizing and consuming enzymes or to supplement NAD synthesis precursors. It has been discovered that there are three major pathways for NAD synthesis: (i) Kynurenine pathway, also called the *de novo* synthesis pathway, in which kynurenine is used as an intermediate to build nicotinic acid carbon ring from tryptophan from the very beginning, and in turn to nicotinic acid mononucleotide (NAMN); (ii) Preiss–Handler pathway, in which the vitamin precursor nicotinic acid (NA) is used to synthesize NAMN first, to the point where two pathways mentioned converge to further synthesize NAD^+^; (iii) Salvage pathway, in which NAD is synthesized from nicotinamide (NAM) or nicotinamide ribose (NR), via a two-step group transfer reaction (Fig. 1A) [7]. Among three pathways, the salvage synthesis pathway is of greater importance for the reason that the product of a wide range of NAD-consuming enzymes, NAM, is available as a substrate for the salvage pathway. Therefore, salvage pathway is more straightforwardly linked to the maintenance of cellular NAD levels. Besides, the related enzymes in the salvage pathway are universally expressed in vivo, unlike the other two pathways, which only undertake the physiological function of synthesizing NAD in a small fraction of tissues.

Therefore, the advantages of using a small molecule activator targeting the rate-limiting enzyme NAMPT in the salvage pathway are: Firstly, compared to other NAD synthetic pathways, the salvage pathway is the major pathway in nearly all cell types. Secondly, compared to supplementing the NAMPT product, NMN, which is still limited by bioavailability and resources, small-molecule activators can more robustly enhance cellular NAD. More importantly, the pharmacokinetics of NAD precursor NMN are hard to predict due to the widely existing extracellular NMN degrading enzyme and the tissue-specific membrane transporters, not to mention the charge-carrying properties of NMN. Thirdly, on a practical level, small molecule activators can be easily synthesized and have more potential for structural optimization in drug development or modification as tool molecules for research. Thus, a series of small molecules targeting NAMPT has been discovered using different paradigms [8].

On the belief of NAMPT’s important role in neuroprotection through the NAD salvage pathway, discovering novel small molecules directly targeting NAMPT to promote NAD synthesis should achieve similar phenotypic results as P7C3, while providing a more diverse toolbox for neurodegeneration-related studies. Thus, a target-based screen on NAMPT was conducted by an *in vitro* coupled fluorescent biochemical reaction. A small molecule that robustly and specifically augments NAMPT enzyme activity was identified and named NAT (Fig. 1B). Based on the co-crystal structure of NAT and NAMPT (Fig. 1C), the researchers hypothesized that NAT functions as an allosteric activator of NAMPT and promotes NAMPT enzyme activity by binding and changing the conformation of amino acid residues near the active site [9, 10]. Based on its potential mechanism of activating NAMPT, the investigators further carried out systematic and rationale structural modification and optimization of NAT. An analog with better protein binding potency and subsequent pharmacological activity has been successfully obtained and named NAT-5r (Fig. 1C). NAT-5r succeeded in elevating NAD levels and causing metabolic reprogramming in cells. When it comes to the original purpose to alleviate neurodegenerative disease-related phenotype, NAT and its derivatives were also found to successfully promote proliferation and self-renewal of neural stem cells, by altering the transcriptome. More importantly, it exhibited neuroprotective efficacy in a chemotherapeutic drug-induced peripheral nerve injury (CIPN) mouse model (Fig. 1D). Together, both NAT and P7C3 achieve neuroprotective effects via activating NAMPT, and once more confirm the feasibility of NAMPT as a neuroprotective drug target. Further study will be required to explain the downstream pathway through which NAMPT activation confers neuroprotection *in vivo*.

NAMPT activators have already shown a broad spectrum of potential in preclinical animal models. Upon its discovery, P7C3 has been shown to alleviate or delay the onset of PD, AD, ALS, and traumatic brain injury [7]. Based on Yao et al.’s preclinical research, it is not hard to speculate that NAT will also show clinical value in the treatment of a series of neurodegenerative diseases as well as aging-related diseases. Still, following studies on NATs’ should take specific clinical models into consideration. Besides, as an exogenous compound, there might be possible off-target effects. Thus, before bench to bedside, NAT’s safety profile should be carefully checked.

In conclusion, two types of small molecules targeting NAMPT have been found to be neuroprotective using both empirical and molecular approaches. The P7C3 chemicals were first derived from phenotypic screening based on anti-neurodevelopmental abnormalities. The unbiased identification of the molecular mechanism of action (MOA) of P7C3 led to the next target-based screening based on NAD salvage pathway enzyme NAMPT, resulting in the discovery of NATs. Although different screening perspectives were adopted, both types of small molecules achieved anti-neurodegenerative effects through the same target NAMPT and NAD biosynthetic pathways, serving as fundamentals for developing new drugs fighting against neurodegenerative diseases.
